# Majority scoring with backward elimination in PLS for high dimensional spectrum data

**DOI:** 10.1038/s41598-021-96389-2

**Published:** 2021-08-20

**Authors:** Freeh N. Alenezi

**Affiliations:** Mathematics Department, College of Science in Zulfi, Majmaah University, Al Majma’ah, 11952 Saudi Arabia

**Keywords:** Cheminformatics, Computational chemistry

## Abstract

Variable selection is crucial issue for high dimensional data modeling, where sample size is smaller compared to number of variables. Recently, majority scoring of filter measures in PLS (MS-PLS) is introduced for variable selection in high dimensional data. Filter measures are not greedy for optimal performance, hence we have proposed majority scoring with backward elimination in PLS (MSBE-PLS). In MSBE-PLS we have considered variable importance on projection (VIP) and selectivity ratio (SR). In each iteration of backward elimination in PLS variables are considered influential if they were selected by both filter indicator. The proposed method is implemented for corn’s and diesel’s content prediction. The corn contents include protein, oil, starch and moisture while diesel contents include boiling point at 50% recovery, cetane number, density, freezing temperature of the fuel, total aromatics, and viscosity. The proposed method outperforms in terms of RMSE when compared with reference methods. In addition to validating the spectrum models, data properties are also examined for explaining prediction behaviors. Moreover, MSBE-PLS select the moderate number of influential variables, hence it presents the parsimonious model for predicting contents based on spectrum data.

## Introduction

For modeling high dimensional data partial least squares (PLS)^1^ has proven itself as potential candidate in diverse areas^2^. PLS is an iterative way of model fitting, where in each iteration PLS components describe the relation between corn’s contents marked as response $\varvec{y}$ and spectrum data marked as explanatory variables $\varvec{X}$. Since PLS is not a method for variable selection, hence several modifications are proposed in PLS for variable selection^3^. The presence of noise variable in high-dimensional spectrum data is quite common, which may affect the prediction capabilities of the model. Although the basic PLS was not designed for variable selection, several developments are made in PLS which accomplish the variable section for improved prediction. Among several developments in PLS the Hotelling $T^2$ based PLS i.e. $T^2$-PLS and truncation on PLS loading weights i.e. Trunc-PLS are considered as potential. The importance of variables in PLS is defined by PLS loading weights. For instance Liland et al.^4^ in Trunc-PLS assumes the normality of loading weights where a set of variables departed from the mean of loading weight’s distribution are considered as noise variables and are discarded from the final fitted model. $T^2$-PLS^5^ can be considered as the multivariate extension of Trunc-PLS, where PLS loading weight matrix having loading weights from first components to optimum components is monitored with Hotelling $T^2$.

Recently, Freeh and Mehmood^6^ has introduced the majority scoring based algorithm for variable selection in PLS (MS-PLS). In MS-PLS several filter measures are considered at same time where variables were scored through considered filter measures. The set of variables which were scored higher compared to threshold were marked as influential and rest were marked as non-influential variables. Mehmood et al.^3,7^ has compared filter and wrapper PLS methods for variable selection in PLS, indicating filter measures are faster while wrapper algorithm are computationally expensive but are more greedy for model performance. Backward elimination procedure is a potential wrapper variable selection method. The current article proposed the implementation of majority scoring in backward elimination, where two variable selection measures are used, variable importance on projection (VIP)^8^ and selectivity ratio (SR)^9^. In each iteration of backward elimination in PLS variables are considered influential if they were selected by both filter indicator. As a case study, the proposed method is implemented for modeling corn contents and diesel contents where samples were characterized by spectrum. The performance of proposed method i.e. MSBE-PLS are compared with reference methods i.e. $T^2$-PLS and Trunc-PLS. In addition to validating the spectrum models, data properties are also examined for explaining corn content’s prediction.

In this paper, “Data set and spectrometers” presents spectroscopic data. “Methods” presents methodology including the PLS based models, parameter estimation, calibration, validation, and statistical analysis. “Results and discussion” presents the results and discussions.

## Data set and spectrometers

We have considered the following two data sets.

### Corn data

The corn Near-infrared spectra samples were obtained from http://software.eigenvector.com/Data/Corn/index.html. The corn data includes 80 samples which were measured on NIR spectrometer called Mp5, which is primary instrument FOSS NIRsystems 5000. The spectrum obtained covers the wavelength range ( 1100 to 2498 nm) at 2 nm intervals having 700 channels per wavelength. This constitute the 700 columns of explanatory matrix. This results in explanatory matrix $X_{(80 \times 700)}$. From each corn sample different contents like protein, oil, starch and moisture were measured. These contents construct the response variables $y_{moisture(80 \times 1)}$, $y_{oil(80 \times 1)}$,$y_{protein(80 \times 1)}$ and $y_{starch(80 \times 1)}$.

### Diesel data

The diesel Near-infrared spectra sampels were obtained from http://www.eigenvector.com/data/SWRI/index.html. The diesel data includes 784 samples. The spectrum covers the wavelength range ( 1100 to 2698 nm) at 4 nm intervals having 401 channels per wavelength. This constitute the 401 columns of explanatory matrix. This results in explanatory matrix $X_{(784 \times 401)}$. From each diesel sample physical properties like boiling point at 50% recovery, cetane number, density, freezing temperature of the fuel, total aromatics, and viscosity were measured. These contents construct the response variables $y_{boiling(784 \times 1)}$, $y_{cetane(784 \times 1)}$, $y_{density(784 \times 1)}$,$y_{freezing(784 \times 1)}$,$y_{aromatics(784 \times 1)}$ and $y_{viscosity(784 \times 1)}$.

## Methods

### Partial least squares (PLS)

In PLS the centered spectrum explanatory matrix $\varvec{X}_{0}=\varvec{X}-\varvec{1}\bar{\varvec{x}}^\prime$ and response $\varvec{y}_{0}=\varvec{y}-1{\bar{y}}$ are used^10^. PLS is an iterative procedure, so it has $K$ components. For all PLS components $k=1, 2, \ldots , K$ the loading weights, score vector, loadings and deflated data are computed as

1. Defining the loading weights by
  $$\begin{aligned} \varvec{w}_{k}=\varvec{X}_{k-1}{^\prime}\varvec{y}_{k-1} \end{aligned}$$
  which reflects the covariance of $\varvec{X}_{k-1}$ with $\varvec{y}_{k-1}$. Normalizing the loading weights
  $$\begin{aligned} \varvec{w}_{k} \leftarrow \varvec{w}_{k}/||\varvec{w}_{k}|| \end{aligned}$$
2. Computing the score vector $\varvec{t}_{k}$ by
  $$\begin{aligned} \varvec{t}_{k} = \varvec{X}_{k-1}\varvec{w}_{k} \end{aligned}$$
3. Computing the X-loading $\varvec{p}_{k}$ through regressing $\varvec{X}_{k-1}$ on the score vector:
  $$\begin{aligned} \varvec{p}_{k}=\varvec{X}_{k-1}{^\prime}\frac{\varvec{t}_{k}}{\varvec{t}_{k}{^\prime}\varvec{t}_{k}} \end{aligned}$$
  Similarly computing the Y-loading $q_{k}$ through
  $$\begin{aligned} q_{k}=\varvec{y}_{k-1}{^\prime}\frac{\varvec{t}_{k}}{\varvec{t}_{k}{^\prime}\varvec{t}_{k}} \end{aligned}$$
4. Deflating $\varvec{X}_{k-1}$ and $\varvec{y}_{k-1}$ by subtracting the involvement of $\varvec{t}_{k}$:
  $$\begin{aligned} \varvec{X}_{k} = \varvec{X}_{k-1}-\varvec{t}_{k}\varvec{p}_{k}{^\prime}\\ \varvec{y}_{k} = \varvec{y}_{k-1}-\varvec{t}_{k}q_{k} \end{aligned}$$
5. If $k<K$ go back to 1.

**Figure 1:**
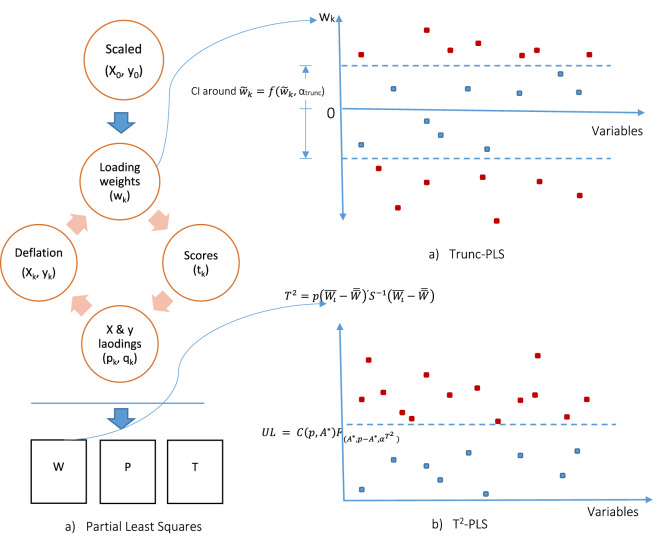
The computational structure of Trunc-PLS and $T^2$-PLS is presented in upper and lower panel respectively.

**Figure 2:**
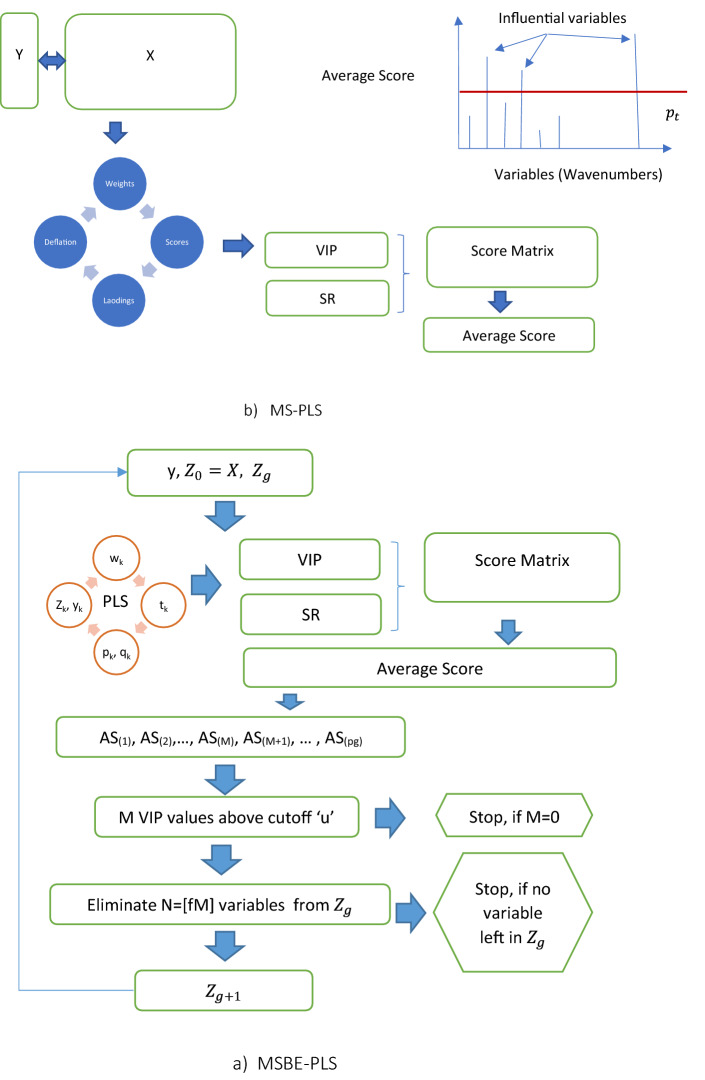
The computational structure of MS-PLS and MSBE-PLS is presented in upper and lower panel respectively.

From each component computed loading weights, score vector, loadings and deflated data is stored in respective matrices/vectors $\varvec{W}$, $\varvec{T}$, $\varvec{P}$ and $\varvec{q}$. Although PLS is suitable candidate for validation, but in presence of noise variables the validation performance may decrease. The validation performance can be improved by removing the noise variables from PLS. We have considered majority scoring backward elimination in PLS (MSBE-PLS), majority scoring in PLS (MSBE-PLS), Hotelling $T^2$ based variable selections in PLS ($T^2$-PLS) and truncation for variable selection in PLS ($Trun$-PLS for modeling corn data. The computational structure of these methods is presented Figs. 1 and 2. The algorithm of these methods is described below.

### Truncation for variable selection in PLS (Trunc-PLS)

In standard PLS, loading weights reflects the importance of variables^11–13^. Variables with small absolute loading weights are considered as noise and should be removed from the model. Considering the importance of PLS loading weights, Liland et al.^4^ assume the PLS loading weight $\varvec{w}$ assumed to follow the normal distribution, where variables located at the tail of normal distribution should be discarded from the model. The procedures follows by:

1. Sorting the PLS loading weights as $\varvec{w_s}$
2. Computing the confidence interval about the median of $\varvec{w_s}$ as $f(\varvec{w_s},\alpha _{Trunc})$.
3. Classifying the outliers as influential variables and inlier as non influential variable.
4. Truncating the non influential variables.

The confidence interval around the median of $\varvec{w_s}$ is dependent over the parameter $\alpha _{Trunc}$, which need to tune for fitting the model. Its higher value indicates lower variables are influential.

### Hotelling $T^2$ based variable selections in PLS $T^2$-PLS

$T^2$-PLS is derived from loading weight matrix $\varvec{W}$. Here Hotelling $T^2$ extracted by $\varvec{W}$ which is assumed to follow the F distribution. The Hotelling $T^2$ measure falls within a certain range are marked as non informative variable that is wavenumber^5^. The algorithm follows.

1. Extract PLS loading weights matrix $W$
2. Translate the PLS loading weights matrix $W{^\prime}$ into Hotelling $T^2$
  $$\begin{aligned} T^2=p({\bar{W}}_i-\bar{{\bar{W}}}){^\prime}S_W^{-1}({\bar{W}}_i-\bar{{\bar{W}}}) \end{aligned}$$
3. Extract the threshold for grouping wavenumbers as influential and non influential variables by
  $$\begin{aligned} Upper limit= C(p,A^*)F_{(A^*, p-A^*, \alpha _{T^2})} \end{aligned}$$
4. Eliminate the non influential variable which fall below the above threshold.

In $T^2$-PLS wavenumber selection is defined by the upper limits $C(p,A^*)F_{(A^*, p-A^*, \alpha _{T^2})}$ which is dependent over $\alpha _{T^2}$ and is required to tune. Its higher value indicates lower variables are influential.

### Majority scoring in PLS (MS-PLS)

Considering more than one filter measure at a time may results in more consistent variable selection, in this context, recently, Freeh and Mehmood^6^ has introduced the majority scoring based algorithm for variable selection in PLS (MS-PLS). Here we have considered variable importance on projection (VIP)^8^ and selectivity ratio (SR)^9^ which are defined as

$$\begin{aligned} VIP_j = \sqrt{p\sum _{c=1}^{C}[(\varvec{q}_c^2 \varvec{t}_c^{\prime } \varvec{t}_c)(w_{cj}/\Vert \varvec{w}_c\Vert )^2]/\sum _{c=1}^{C}(\varvec{q}_c^2 \varvec{t}_c^{\prime } \varvec{t}_c)}. \end{aligned}$$

For selectivity ratio (S) target projection also called target rotation is used. Target projection is the post projection of explanatory spectrum data on the response that is the antibacterial activity of ILS, where spectrum explanatory matrix is decomposed into the residual part and latent part as

$$\begin{aligned} \varvec{X}=\hat{\varvec{X}}_{TP}+\varvec{E}_{TP}=\varvec{t}_{TP}\varvec{p{^\prime}}_{TP}+\varvec{E}_{TP} \end{aligned}$$

where $\varvec{t}_{TP}=\varvec{Xw}_{TP}$, $\varvec{w}_{TP}=\varvec{{\hat{\beta }}_{PLS}/||{\hat{\beta }}_{PLS}||}$ and $\varvec{p}_{TP}=\varvec{X{^\prime}t}_{TP}/(\varvec{t}_{TP}{^\prime}\varvec{t}_{TP})$. The selectivity ratio (S) from TP defined as

$$\begin{aligned} S_{j}= V_{exp, j}/V_{res,j} \end{aligned}$$

where $V_{exp, j}$ is the explained variance through TP and $V_{res,j}$ is the residual variance of spectrum $j$. The proposed procedure is presented in a flow chart in Fig. 2 and is described as

1. Fit the PLS regression model.
2. Compute the filter measures VIP and SR against all PLS components.
3. Construct the score matrix $S$ whose column presents the variable and rows presents the filter measures. The $(i{\text{th}} row,j{\text{th}} column)$ entry of $S$ matrix presents the influence of $i{\text{th}}$ filter measure over $j{\text{th}}$ variable.
4. Compute the average score ($\psi \in [0,1]$) for each $j{\text{th}}$ variable. ($\psi \rightarrow 1$) indicates respective variable is influential.
5. convert $\psi$ into label vector $l_{\psi }$ as
  $$\begin{aligned} \varvec{l_{\psi }}_{i,j}= {\left\{ \begin{array}{ll} 1,&{} \text {if }\psi _j \ge pt \\ 0, &{} \text {otherwise} \end{array}\right. } \end{aligned}$$
  Here $pt$ is percentile. Its higher level is expected to result in influential variable selection. For optimal performance, it is required to tune.

### Majority scoring with backward elimination in PLS (MSBE-PLS)

Majority scoring with backward elimination in PLS (MSBE-PLS) required the same filter measure as taken in MSBE for variable importance for variable selection. Let $\varvec{Z}_0 = \varvec{X}$ then the procedure follows.

1. Fit the cross validated PLS model on $\varvec{y}$ by $\varvec{Z}_g$ having $p_g$ number of variables.
2. From fitted model extract the $VIP$ and $SR$
3. Find the average score and sort them in ascending order.
4. Against the threshold $u$ on average scores, if there are $M$ variables below the threshold than $N=\lceil fM\rceil$ variables need to removed from the model, where $f\in \langle 0,1]$.
5. In case more than one variable left in the model than move the step 1 else stop the iteration.

The $f$ defines the fraction of removed variables, closer to 0 will remove very few variables and vice versa. We have fixed $f=0.1$ means in each iteration very few variables will be removed. In MSBE-PLS the threshold $u$ needs to tune for model fitting. See the computational structure of MSBE-PLS in Fig. 2.

### Model fitting

Model fitting requires parameter tuning. For all three considered PLS based methods, number of PLS components is common parameter to tune. In addition to this Trunc-PLS has $$\alpha _{Trunc}$$, $$T^2-PLS $$ has $$\alpha _{T^2}$$ and MSBE-PLS has $$u$$. These additional parameters defines the variable selection in respective PLS models. For optimal estimation, a range of possible values of these parameters is considered in validation procedure described in upcoming subsection.

### Validation and robustness of model performance

For evaluating the model prediction capability and reliable estimation of parameters double cross-validation procedure is adapted. The spectrum data $\varvec{X}$ and response $y$ is divided into test (25%) and training $75\%$. The training data is used for model fitting. The prediction capability, which is usually measured by RMSE is defined as

$$\begin{aligned} RMSPE= \sqrt{\frac{\sum _{i=1}^{n}(y_i-{\hat{y}}_i)^2}{n}} \end{aligned}$$

where n sample of respective split of data (test/training), $y_i$ is the response which can be any of the corn or diesel content and ${\hat{y}}_i$ is respective predicted response from the model. The model with lest RMSE on training and test data set is called well calibrated and well validated model respectively. Since model fitting requires the parameter tuning, hence the 10-fold cross validation is used on training data. The parameter threshold which gives the best RMSE in 10 fold cross validation is considered as the optimum.

The data is divided into training and test randomly, hence it quite possible for given split the models may over or under perform. In order to have robust model performance estimation Monte Carlo simulation with 100 runs was used. In each Monte Carlo simulation run, the above procedure of validation is conducted^14^.

### Data properties

In addition, data properties are also examined for explaining corn and diesel content’s prediction. For this purpose, eigenvalue structure of sample covariance spectrum matrix and the covariance between principle components and the contents^4,15^. Irrelevant components having large eigenvalues are expected to have worst prediction.

## Results and discussion

From corn samples four protein, oil, starch and moisture are measured, from diesel boiling point at 50% recovery, cetane number, density, freezing temperature of the fuel, total aromatics, and viscosity are measured. Hence each response is modeled separately with respective spectrum .

**Figure 3:**
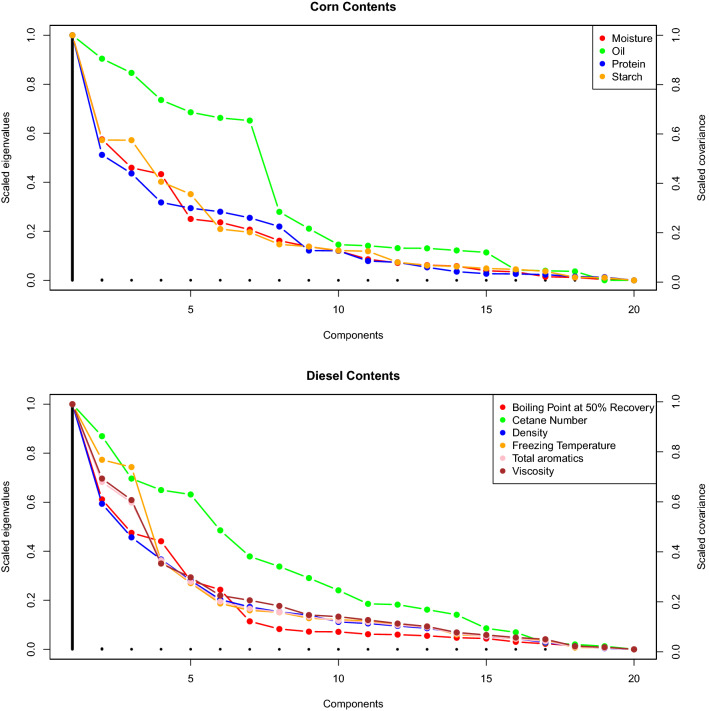
Data properties related to corn and diesel spectrum and their contents are extracted for understanding the prediction behavior. The eigenvalue (sorted in descending order) presents the covariance of spectral matrix. The points indicates the covariance (sorted in depending order) between principal components and each content.

The data properties related to corn and diesel spectrum and their contents are presented in Fig. 3. Upper panel presents, corn spectrum has strong between-variable dependencies. Very few latent components seem to explain most of data variation. Together with the sharp drop of eigenvalues, we notice distinct behavior of spectral covariances between the principal component and corn contents. On the average, moisture and oil show large covariances over the relevant components and small covariances over the irrelevant components, hence one should expect better prediction. Protein shows moderate covariances over the relevant components and small covariances over the irrelevant components, hence one should expect moderate prediction. Starch show small covariances over the relevant and irrelevant components, hence one should expect relatively low prediction. Similar trends are observed with diesel contents as presented in lower panel of Fig. 3.

Since we have considered four PLS based models including Trunc-PLS, $T^2$-PLS, Ms-PLS and MSBE-PLS. For evaluating and comparison Monte Carlo simulation is implemented with $N=100$. In each run, the spectrum data $\varvec{X}$ and contents $y$ are divided into test (25%) and training $75\%$. Training data is used to fit the PLS based model, where 10 fold cross validation is implemented for tuning the model parameters like number of components, $\alpha _{Trunc}$, $\alpha _{T^2}$ and $u$. From each Monte Carlo run optimal tuning parameters, calibration RMSE , validated RMSE and number of selected wavenumbers are recorded for each of the fitted model.

**Figure 4:**
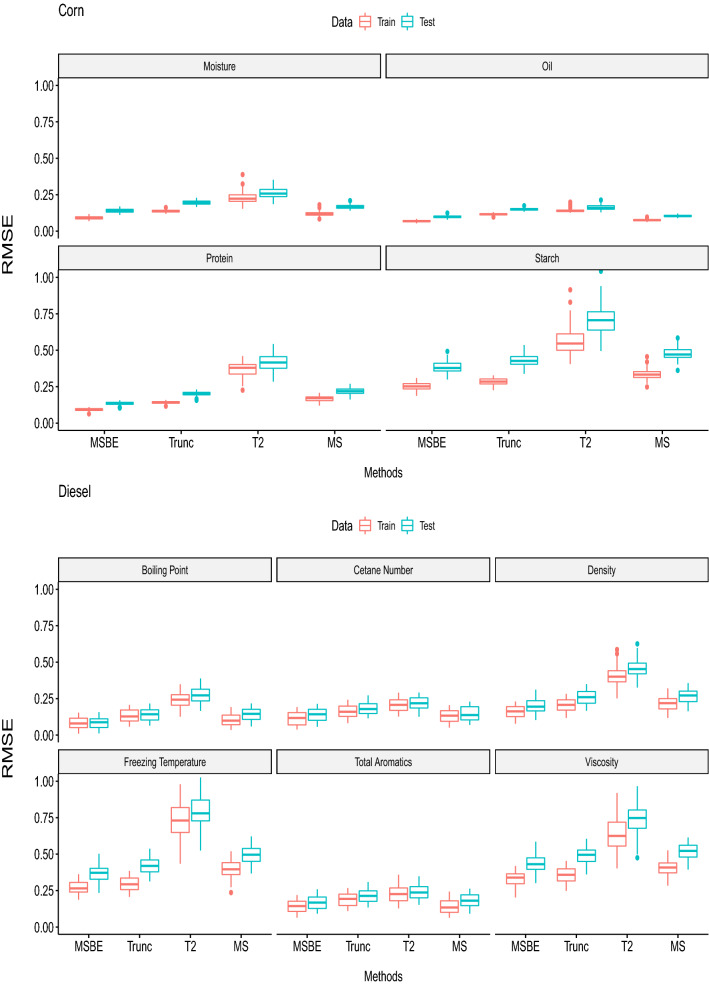
The distribution of train and test RMSEP together with standard error bars is presented for all fitted model of corn and diesel contents. Trends are extracted based on the 100 Monte Carlo simulation.

For prediction models the both validated and calibrated RMSE should be small^16^. The comparison of validated and calibrated RMSE for corn and diesel content is presented in Fig. 4. $$Rep-PLS $$ and Trunc-PLS show small validated and calibrated RMSE. Moreover $$T^2$$-PLS has moderate validated and calibrated RMSE. Similarly, we found the same trend for other corn contents. Generally we can conclude $$T^2$$-PLS has worst validated and calibrated RMSE and MSBE has outperformed the other methods.

**Figure 5:**
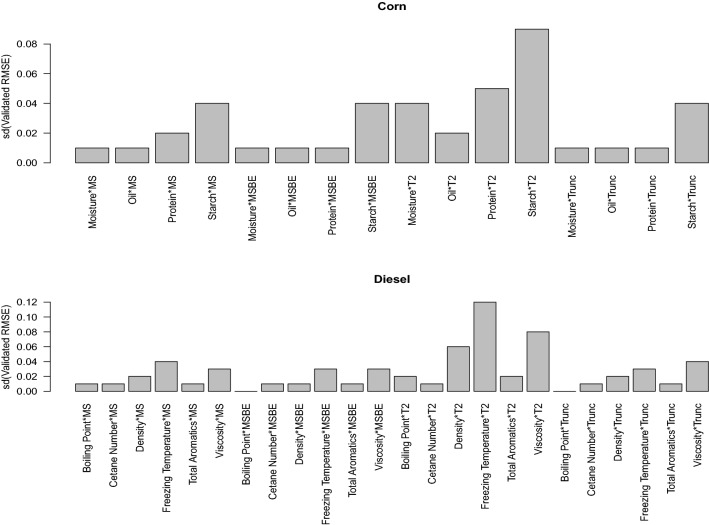
The standard deviations of validated RMSE for all fitted models of corn and diesel contents are presented.

For validation, the stability of the model is also an important factor to consider. In Fig. 5 the standard deviations of accuracy for all fitted model is presented. MS-PLS, MSBE-PLS and Trunc-PLS has the best stability for corn moisture and oil content. MSBE-PLS and Trunc-PLS has better stability for corn protein contents. Similarly, the boiling point of diesel has best stability with MSBE-PLS and Trunc-PLS. The cetane number has good stability with all PLS methods. The diesel density has best stability with MSBE-PLS. The freezing temperature of the fuel has best stability with MSBE-PLS. The total aromatics has best stability with MSBE-PLS and Trunc-PLS. The viscosity has best stability with MSBE-PLS and MS-PLS.

**Figure 6:**
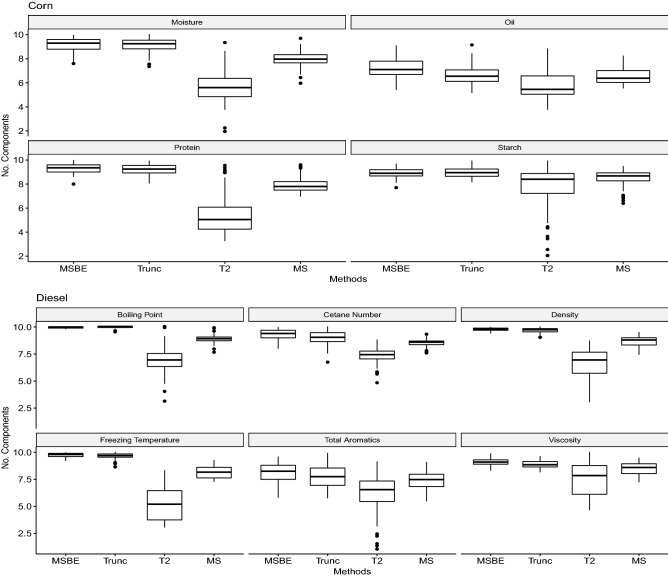
The distribution of number of components presenting the complexity of the model is presented for all fitted corn and diesel contents models. Trends are extracted based on the 100 Monte Carlo simulation.

After conducting the validated and calibrated RMSE comparison, and stability analysis. Analysis of variance (ANOVA) is conducted to study the effect of validation methods over the variations in validated RMSE. The ANOVA results for each corn characteristic protein, oil, starch and moisture are presented in Table 1. Among PLS models MSBE-PLS is taken as a reference model. It appears MSBE-PLS has significantly better prediction of corn’s moisture (p-value=0.018) and oil (p-value $<0.001$) compared to Trunc-PLS, similarly MSBE-PLS has significantly better prediction of all considered corn’s contents (p-value $<0.001$) compared to $T^2$-PLS. The ANOVA results for each diesel characteristic diesel boiling point at 50% recovery, cetane number, density, freezing temperature of the fuel, total aromatics, and viscosity are presented in Table 2. It appears MSBE-PLS has significantly better prediction of diesel’s content (p-value=0.018). The complexity of the model is usually defined by the number of PLS components. The distribution of number of components presenting the complexity of the model is presented in Fig. 6 for all fitted corn and diesel contents models. Trends are extracted based on the 100 Monte Carlo simulation. Results indicates, for corn’s moisture MSBE-PLS and Trunc-PLS consumes larger number of PLS components and are considered as complex model. For corn’s oil all methods consumes moderate number of PLS components. For corn’s protein MSBE-PLS and Trunc-PLS consumes larger number of PLS components and are considered as complex model. For corn’s starch all consumes larger number of PLS components and are considered as complex model. Similarly for diesel contents modeling most of the PLS models are complex as they consumes larger number of components and $T^2$-PLS which consumes moderate number of components.

**Table 1:** The ANOVA results indicating the significant PLS model against each corn characteristic moisture, oil, protein, and starch are presented.

Factor	Level	Moisture		Oil		Protein		Starch	
		Estimate	P-value	Estimate	P-value	Estimate	P-value	Estimate	P-value
Intercept		0.007	< 0.001	0.063	< 0.001	0.126	< 0.001	0.297	< 0.001
Model	$MSBE-PLS$	Reference							
	$MS-PLS$	0.035	0.021	0.034	< 0.001	0.035	0.014	0.026	0.081
	Trunc-PLS	0.007	0.018	0.004	< 0.001	0.006	0.139	-0.006	0.481
	$T^2$-PLS	0.144	< 0.001	0.026	< 0.001	0.201	< 0.001	0.376	< 0.001

**Table 2:** The ANOVA results indicating the significant PLS model against each diesel characteristic boiling point at 50% recovery, cetane number, density, freezing temperature of the fuel, total aromatics, and viscosity are presented.

Model Level	Boiling point		Cetane Number		Density		Freezing temperature		Total aromatics		Viscosity	
Model	Estimate	P-value	Estimate	P-value	Estimate	P-value	Estimate	P-value	Estimate	P-value	Estimate	P-value
Intercept	0.012	< 0.001	0.094	< 0.001	0.378	< 0.001	0.304	< 0.001	0.218	< 0.001	0.245	< 0.001
$MSBE-PLS$	Reference											
$MS-PLS$	0.147	0.034	0.025	< 0.001	0.087	0.021	0.036	0.029	0.247	< 0.001	0.654	< 0.001
Trunc-PLS	0.013	0.045	0.021	< 0.001	0.054	0.297	0.012	0.274	0.214	< 0.001	0.301	< 0.001
$T^2$-PLS	0.614	< 0.001	0.158	< 0.001	0.413	< 0.001	0.207	< 0.001	0.314	< 0.001	0.245	< 0.001

**Figure 7:**
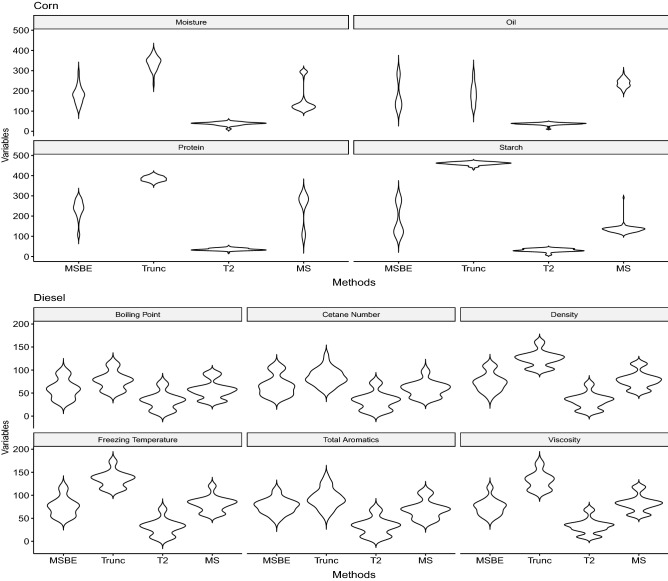
The distribution of number of selected variables i.e. wavenumbers is presented for all fitted corn and diesel contents models. Trends are extracted based on the 100 Monte Carlo simulation.

For a well calibrated and validated model the number of selected variables is important to consider since it reflect how much information is considered noise and how much information is considered influential. Moreover the distribution of selected number of variables effects the prediction that RMSE. The distribution of number of selected variables together with standard error bars from 100 Monte Carlo simulation is presented for all of PLS methods in Fig. 7. The upper panel presents the distribution of selected variables in modeling the corn’s contents while lower panel presents the results for diesel contents. It appears, Trunc-PLS is using the maximum number of variables (wavelength) while $T^2$-PLS utilizes the minimum number of variables. Since the prediction capabilities from Fig. 6 shows MSBE-PLS and Trunc-PLS have better prediction capability, hence the parsimonious corn’s moisture modeling can be achieved with MSBE-PLS. By parsimonious model we mean the better prediction with the least number of selected variables. The distribution of selected variables in modeling the corn’s oil indicates MSBE-PLS and Trunc-PLS are based on an almost equal number of variables, while $T^2$-PLS is based on the least number of variables. The distribution of selected variables in modeling the corn’s protein indicates the MSBE-PLS and Trunc-PLS are based on almost equal number of variables, while $T^2$-PLS is based on the least number of variables. Figures 6 and 7 shows MSBE-PLS both can be used to model the corn’s protein irrespective of any spectrum being used. The behavior of MSBE-PLS is expected because of the architecture of the algorithm^17^. The distribution of selected variables in modeling the corn’s starch indicates MSBE-PLS and Trunc-PLS are based on an almost equal number of variables, while $T^2$-PLS is again based on the least number of variables. Similar trend are observed while modeling the diesel contents.

## Conclusion

PLS based validated model is proposed for variable selection of spectrum data. The corn and diesel content are characterised based on eigenvalue and covariance between principal components and response. Results from Monte Carlo simulation reveals MSBE-PLS has both smallest validated and calibrated RMSE for all corn’s and diesel contents. On the average, all considered PLS based methods and all spectrometers has significantly different prediction (p-value=0.001). In terms of prediction MSBE-PLS and Trunc-PLS are better compared to $T^2$-PLS, while $T^2$-PLS has small, MSBE-PLS has moderate and Trunc-PLS has large number influential variables. Hence, MSBE-PLS is the parsimonious model for predicting.
